# Biophysical Study of the Structure, Dynamics, and Function of Nucleic Acids

**DOI:** 10.3390/ijms23105836

**Published:** 2022-05-23

**Authors:** Joon-Hwa Lee, Masato Katahira

**Affiliations:** 1 Department of Chemistry and RINS, Gyeongsang National University, Jinju 52828, Gyeongnam, Korea; 2 Institute of Advanced Energy, Kyoto University, Uji 611-0011, Kyoto, Japan

Nucleic acids have essential roles in all biological processes related to genetic information, such as replication, transcription, translation, repair, and recombination. Over the years, biophysical tools such as X-ray crystallography, NMR spectroscopy, and cryo-EM have been employed to study nucleic acids’ structure and understand their biological functions. In addition, biophysical studies of the dynamic features of nucleic acids should be conducted because their less populated conformations can contribute to folding, stability, and biological functions. Recently, many experimental and theoretical approaches have been reported to understand the correlation between the structure and dynamics of nucleic acids and their biological functions.

This Special Issue in the International Journal of Molecular Sciences, entitled “Biophysical Study of the Structure, Dynamics, and Function of Nucleic Acids”, contains 14 invited research articles. G-quadruplex (G4) is a non-canonical structure produced in the guanine-rich (G-rich) regions of nucleic acids and is essential for its diverse functions during various biological processes. Here, Pagano et al. performed the biophysical and biochemical studies on the AS1411 derivatives differing in the sequence length and base composition from AS1411, a G4-forming G-rich oligonucleotide [1]. This study reported that AS1411 derivatives have high inhibitory effects against topoisomerase I and improved antiproliferative activity on MCF cells due to the multi-targeted effects [1]. Katahira group investigated the intermolecular interaction of the part of human origin recognition complex subunit 1 (hORC1^413–511^) with various G4-forming G-rich DNAs [2]. This study revealed that hORC1^413–511^ recognized the external G4-tetrad planes of the G4 structure of DNA [2].

This Special Issue contains three research articles that studied nucleic acid methylation, a prevalent regulatory modification in *prokaryotes* and *eukaryotes*. Tsuruta et al. investigated the methylation effect within the CpG site on DNA duplexes’ thermodynamics under cell-mimicking molecular crowding conditions [3]. Lam et al. revealed that the methylated adenine base, such as m^1^A and m^6^A, formed the Hoogsteen base pairs with the opposite T bases in a mini-dumbbell structure of two TTTA tetranucleotide repeats [4]. Yoneda, Ueda, and Kurokawa investigated the binding specificity of a nuclear RNA binding protein, Translocated in LipoSarcomar/Fused in Sarcoma (TLS/FUS), to m^6^A-modified RNA fragments and proposed that TLS/FUS is a novel candidate for m^6^A recognition protein [5].

Two articles reported the synthesis and biological application of the chemically modified oligonucleotides in this issue. Novopashina et al. synthesized the nucleotide conjugates of *closo*-dodecaborate attached to 5′-, 3′-, or both terminal positions of DNA, RNA, 2′-OMe RNA, and 2′-F RNA [6]. This study found that the incorporation of terminal boron clusters into the oligonucleotides caused the increase in hydrophobicity, change in thermal stability of duplexes, and the B-to-A structural change in DNA conjugates [6]. Taniguchi et al. demonstrated that the 7,8-dihalogenated 7-deaza-dGTP showed a strong inhibitory effect against MTH1, which hydrolyzed an oxidatively damaged 8-oxo-dGTP [7].

This issue contains four research articles that studied the biological function and structural dynamics of non-coding RNAs. Using NMR spectroscopy, Choi et al. reported a base-pair opening dynamics study of Bacillus cereus fluoride riboswitch [8]. The authors demonstrated that the binding of fluoride anion led to great stabilization of the U45·A37 base pair without the conformational change in riboswitch and resulted in transcription regulation [8]. Ohyama group demonstrated that a cluster of cruciform formable inverted repeats (CFIRs), found within the mouse *Pou5f1* enhancer, played an active role in the transcriptional regulation of *Pou5f1*, as well as *Sox2*, *Nanog*, *Klf4*, and *Esrrb* [9]. Sato et al. showed that the packaging signal (psi) RNA from the HIV-1 regional variant (subtype D) had a low packaging ability compared with another variant (subtype B) [10]. This study revealed that the psi 226/227 dinucleotide pair functions as a cis-acting regulator to control the psi structure to selectively tune the efficiency of packaging, but not dimerization of highly variable HIV-1 genomes [10]. Westerlund et al. demonstrated that pre-miRNA-377 exhibited the folding heterogeneity originating from the large size of the hairpin loop, whose mechanical stability was able to be enhanced by the recently developed C2 ligand [11].

Three original articles studied the dynamics of DNA and DNA–protein complexes that contributed to this Special Issue. Lee et al. developed a new global search algorithm to elucidate the dynamics properties in the DNA binding of proteins using Carr–Purcell–Meiboom–Gill relaxation dispersion data [12]. The global search performed by the authors indicated that an α-helical segment of the Zα domain of human ADAR1 (hZα_ADAR1_) provided the main contribution to the three-state conformational changes in a hZα_ADAR1_–DNA complex with a slow B–Z exchange process [12]. Viader-Godoy et al. reported the pulling experiments with optical tweezers to characterize the elastic response of ssDNA over three orders of magnitude in length (60–14 k bases) [13]. They demonstrated that the different force regimes fitted for long and short molecules interpreted a force-induced sugar pucker conformational transition (*C3′-endo* to *C2′-endo*) upon pulling ssDNA [13]. Dzhimak group investigated the effect of various frequencies of external periodic action in the range from 10^11^ s^−1^ to 10^8^ s^−1^ on the dynamics of a DNA molecule and found that under the influence of an external periodic force, a DNA molecule exhibited oscillatory movements with a specific frequency characteristic of this molecule, which depended on the sequence of nucleotides [14].

Overall, the 14 contributions that make up this Special Issue highlight the interesting structural features and unusual biological functions of nucleic acids. Finally, we would like to express my heartfelt gratitude to all of the authors and referees for their tremendous and relentless efforts in supporting this Special Issue. Without their valuable assistance, we would not have glanced at this successful publication on the Biophysical Study of the Structure, Dynamics, and Function of Nucleic Acids.
